# Advanced molecular approaches to thalassemia disorder and the selection of molecular-level diagnostic testing in resource-limited settings

**DOI:** 10.1016/j.htct.2025.103860

**Published:** 2025-06-14

**Authors:** Balaiah Meenakumari, Chandramouleeswari K, Sariga Dhanasekar

**Affiliations:** Institute of Child health and hospital for children, Rina mandal road, Egmore, Chennai, Tamil Nadu, India

**Keywords:** Thalassemia, QPCR, Epigenetics, Gene editing, Sequencing

## Abstract

Beta-thalassemia is a genetic disorder that significantly burdens healthcare systems globally. This inherited blood disorder, categorized into beta-thalassemia and alpha-thalassemia, results in insufficient globin production, leading to anemia and iron overload from frequent transfusions. Severe cases, known as thalassemia major, require regular blood transfusions. Beyond clinical suspicion and biochemical tests, molecular techniques are essential for confirming the diagnosis and guiding treatment. Advanced molecular profiling methods such as Polymerase Chain Reaction (PCR), Multiplex Ligation-dependent Probe Amplification (MLPA), Next-Generation Sequencing (NGS), Third-Generation Sequencing (TGS), and Clustered Regularly Interspaced Short Palindromic Repeats (CRISPR) are effective in detecting mutations. Epigenetic factors also play a crucial role, driving the development of epidrugs for targeted therapy. This review covers various molecular techniques, established gene-editing methods, epigenetic mechanisms, and the impact of artificial intelligence on thalassemia management. It highlights the importance of selecting precise and sensitive molecular tools for detecting thalassemia gene mutations and stresses the need to make these testing methods accessible in resource-limited clinical settings.

## Introduction

Thalassemia, a monogenic disorder among hemoglobinopathies, has a universally recessive inheritance affecting both children and adults worldwide. In India, beta-thalassemia accounts for 25 % of the global burden.^1^ Beta-thalassemia is particularly prevalent in Mediterranean countries, Middle East, and South Asia, regions historically affected by malaria. In India, the highest prevalence is found in the northern states of Punjab, Haryana, and Delhi, and the western states of Maharashtra and Gujarat, with the lowest prevalence in the southern states of Tamil Nadu and Karnataka.^2^^,^^3^ The challenges faced by the patients and their families due to this disease are substantial. Affected individuals require lifelong regular blood transfusions and chelation therapy, leading to complications such as heart disease, liver damage, and endocrine disorders.^4^ In rural areas, the cost of the treatment, medical care and testing services pose a significant constraint. In urban areas, too, the disease burden significantly impacts the health system and resources.

Thalassemias are clinically categorized as thalassemia major (TM), thalassemia intermedia (TI) and thalassemia minor or trait, based on the severity. TM is the most serious form, requiring regular treatment. Thalassemia is classified into two based on the globin gene defect: alpha-thalassemia (Hemoglobin Subunit Alpha 1: *HBA1* and Hemoglobin Subunit Alpha 2: *HBA2* genes) and beta-thalassemia (Hemoglobin Subunit Beta: *HBB* gene). In beta-thalassemia, substitutions of bases occur in the introns, exons and promotor regions of the beta-globin genes, whereas in alpha-thalassemia, base deletions lead to the removal of alpha genes.^5^ The alpha gene is located on chromosome 16p13.3, and the beta gene is clustered among other hemoglobin genes on chromosome 11p15.15.

The diagnosis and detection of thalassemia involve several laboratory examinations such as complete blood count, blood smear, iron studies, hemoglobinopathy studies, DNA analysis by genetic testing, and prenatal genetic testing.^6^ Based on the clinical, hematological and molecular features, beta-thalassemia is categorized into two distinct types based on blood transfusion: non-transfusion-dependent β-thalassemia (NTDT), which is TI, and transfusion-dependent β-thalassemia (TDT), which is TM. Preliminary screening methodologies are economical and feasible for mass coverage of the disease-causing genes in the society, helpful in triaging patients who require a DNA analysis through superior and high-throughput technology. However, in routine clinical practice, mutation testing for these genes is not commonly practiced. Instead, driven by market forces, patients are often directly referred for NGS testing assuring one-stop solutions. Therefore, it is advocated that discussions between clinical genetic departments and diagnosticians should prioritize less expensive methodologies with superior specificity and reliability in terms of test quality to triage patients and effectively utilize NGS technology.^7^ Hence this review aims to study different molecular methodologies and high-throughput tests affecting the detection level of thalassemia, a hematological disorder of high societal impact, and its future implications in clinical practice.

## Molecular profiling of thalassemia

**Figure 1:**
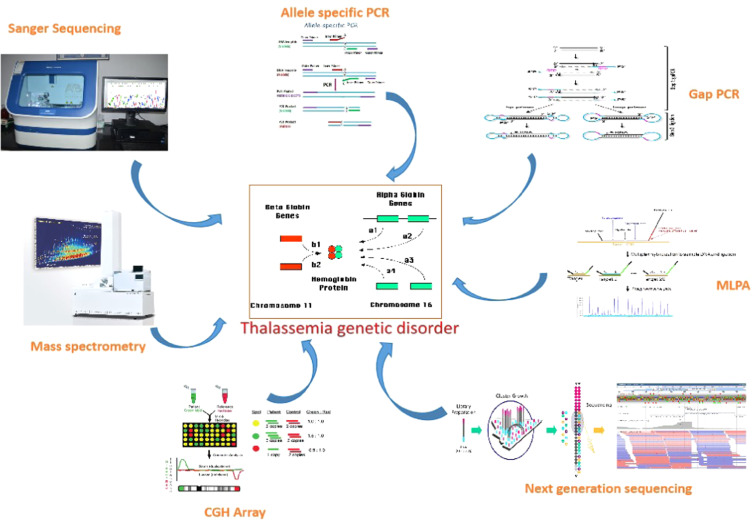
Illustration of the different molecular tools for diagnosis of thalassemia.

Various molecular profiling methods exist for diagnosis, each with its limitations. The available molecular genetic testing for thalassemia is single gene testing.^8^ For beta-thalassemia, *HBB* gene sequencing analysis is offered to detect mutations. However, due to the identical length of the *HBA1* and *HBA2* genes, sequencing analysis for alpha-thalassemia has been challenging.^9^ Protein-based detection methods such as electrophoresis and chromatography are commonly used in routine practice. To prevent adverse outcomes of globin genetic disorders, along with genetic confirmation in a given patient, genetic testing is important for potential carriers in prenatal and premarital contexts.^10^ The list of molecular techniques used for detecting thalassemia is summarized in Table 1 and Figure 1.

**Table 1:** List of molecular techniques, their application and disadvantages.

Technique	Application	Disadvantages
Sanger sequencing	Detects all possible mutations in an individual.	Not useful for detecting large deletions
Allele-specific methodologies (allele-specific polymerase chain reaction)	Useful in genetically homogeneous populations, high throughput and economical	Less useful in ethnically diverse populations
Gap-Polymerase Chain Reaction	Rapid and multiplexed	Cannot detect point mutations, requires specific primers
Multiplex ligase-dependent probe amplification (MLPA)	Can cover large chromosomal regions for deletion analysis	Low resolution, cannot detect point mutations or small deletions
Next-generation sequencing (NGS)	Has potential to characterize mutations and deletions throughout all globin genes in parallel	Needs to create awareness about the technique
Comparative genomic hybridization (CGH)	covers large chromosomal regions for deletion analysis, high resolution and has exact breakpoints	Not reliable due to cross-hybridization
Mass spectrometry	Hemoglobin variants can be characterized rapidly	Yet to be approved in routine diagnostics
Artificial Intelligence (AI)	Differentiates between thalassemia and other microcytic anemia by using different algorithms and web-based prediction tools.	Not yet approved in routine diagnostics

### Recent molecular approaches

Advancements in techniques for detecting thalassemia include NGS, which can accurately distinguish rare mutations and reduce misdiagnoses. Extensive work has been conducted on alpha- and beta-thalassemia mutation screening using NGS technology over the past few years. While whole genome sequencing, exome sequencing, RNA sequencing, and methylation sequencing are widely used NGS applications, targeted sequencing is the most effective and economical approach for thalassemia, covering indels and point mutations in the *HBA, HBB,* Hemoglobin Subunit Delta (*HBD*) and Hemoglobin Subunit Gamma (*HBG*) genes.^11^ Conventional methods only detect specific mutations targeted by primer sets, but NGS provides a more extensive and thorough analysis of the individual’s genetic make-up in a single test and can detect multiple mutations from a single gene or multiple genes. NGS is more reliable in characterizing the disease genotype, and its deep sequencing is used to identify mutations in diagnosing many human genetic disorders.^12^

Recently, Gupta et al. developed a scalable non-invasive amplicon-based precision sequencing (SNAPseq) assay system, a unique strategy-based NGS approach to detect virtually all *HBB* mutations. The SNAPseq assay utilizes a simple, extraction-free non-invasive buccal swab crude lysate or finger prick blood sample directly for detecting allele-specific beta-thalassemia and sickle cell disorder genotypes. Their study showed a simplified sampling procedure combined with an NGS approach to develop and optimize pipelines to prioritize pathogenic mutations with allele-specific sensitivity. They concluded that their assay could serve as a gold standard technique applicable for precise diagnosis of beta-hemoglobinopathies with high sensitivity.^13^

Third-Generation Sequencing (TGS) - the next era of DNA sequencing technology, has gained prominence in molecular biology, studying genomes, transcriptomes, and metagenomes without the need for clonal amplification. Oxford Nanopore Technology (OCT) and the Pac-Bio Single Molecule Real-Time Sequencing (SMRT) are the two TGS technologies currently available. The major challenge in TGS is the accurate identification of the nucleotide bases due to the instability of the molecular machinery involved, resulting in higher error rates than NGS. Several studies have clinically utilized the TGS approach to identify both alpha- and beta-thalassemia genetic carrier statuses, with results showing complete concordance with conventional molecular techniques.^14^^,^^15^ A study conducted by Zhen-min et al.^17^ reported rare mutations in *HBA, HBB, HBD*, and Hemoglobin H genes in children with mild anemia. They identified rare mutations in children with suspected transfusion-dependent thalassemia (TDT), necessitating long-term blood transfusions using the TSG approach. Zhuang et al.^16^ also reported identifying rare variants in the *HBA* gene by TGS technology. Hence, TSG can serve as a diagnostic tool to effectively screen thalassemia carrier trait in at-risk individuals or couples.^16^, ^17^, ^18^, ^19^

The innovation of the CRISPR-associated protein 9 (CRISPR-Cas9) system, a genome editing technology, revolutionized biomedical research. This system is widely used for DNA base editing, RNA targeting, gene expression regulation and epigenetic editing for preventing and managing various genetic diseases. Though the technology has many challenges, due to its ease of use, higher efficiency, specificity and cost-effectiveness, it is more extensively used than other genome editing techniques.^20^ Current curative stem cell or bone marrow transplantation for thalassemia has the limitation of obtaining an HLA matched donor within the family or an unrelated individual. Graft-versus-host disease and the high cost compared to gene editing make gene editing a potential curative option.^21^ CRISPR-Cas9 gene editing technology is applied to correct the alpha- or beta-globin chain imbalance in thalassemia hematopoietic stem/progenitor cells by down regulating the alpha-globin locus to control *HBB* gene expression.^22^ An editorial by Parums discusses the first regulatory approval for CRISPR-Cas9 gene editing therapy, Casgevy (exagamglogene autotemcel) and Lyfgenia (lovotibeglogene autotemcel), for treating patients with transfusion-dependent beta-thalassemia and sickle cell disease. He discusses the therapeutic challenges and outcomes of patients treated with CRISPR-Cas9 therapy.^23^ The end-point of several clinical trial studies will warrant the treatment management of thalassemia and sickle cell anemia through gene editing therapy. Still, gene editing technology has limitations, which will be overcome by the new prime editing technology.^24^ Advancements in gene editing technology, such as CRISPR, may soon surpass allogeneic transplants as the preferred treatment for patients with sickle cell disease or thalassemia. These cutting-edge techniques offer the potential for more precise and personalized treatments, potentially reducing the risks and complications associated with traditional transplant methods.

### Epigenetic aspect of thalassemia

Developments in the field of medical genetics focus more on the regulatory machinery of gene expression through epigenetics, thereby providing a new entity of therapeutic targets for treating various genetic disorders. The alteration of gene activity without the change in DNA sequence by histone modification and DNA methylation is an epigenetic concept. Epigenetic modifiers play a significant role in alpha- and beta-thalassemia disorders. In alpha-thalassemia, the common mutation types are often deletions affecting one or more of the alpha-globin genes (HBA1 and HBA2) or one pseudogene with a homozygous configuration of the allele, which results in the hydrops fetalis form. The DNA methylation level in association with this mutation results in a differential methylation pattern between placenta and leukocytes.^25^ In beta-thalassemia, the epigenetic modification changes fetal hemoglobin (Hb F) to adult hemoglobin (Hb A). The delay in conversion of Hb F to Hb A is due to the regulatory single nucleotide polymorphism (r-SNP), which leads to clinical complexity of the disease by keeping Hb F levels high. The DNA methylation in beta-thalassemia down regulates the beta-globin gene and up regulates the production of the gamma-globin gene with co-inheritance of alpha-thalassemia, which improves beta-thalassemia severity. The enhancement of gamma-globin gene expression in beta-thalassemia is due to the demethylation of the promotor Cytosine-phosphate-Guanine (CpG) sites in erythroid progenitor cells^26^. In a study by Yassim et al.^27^ it was found that the Immunoglobin superfamily 4 (IGSF4) has an important role in the synthesis of the globin chain. Due to the methylation of *IGFS4,* the synthesis of the globin chain is affected by its interaction with other genes in the regulation network of globin expression.

This disease-causing epigenetic change can be revisited by the use of epigenome editing to control the regulation of gene expression by writing and erasing the epigenetic modifiers. Some of the epigenetic modifiers include DNA modifiers, mRNA modifiers and histone protein modifiers. The IGSF4 and La ribonucleo protein 2 (LARP2) modifiers were hypermethylated in beta-thalassemia major patients.^26^ The development of epigenetic drugs called epidrugs was utilized initially to reverse the nature of epigenetic alterations. Epidrugs target different epigenetic marks and inhibit disease-causing alterations. Their effect is not sequence-specific and can lead to cell death due to a broad alteration of gene expression.^28^ To assuage this effect, epigenome editing technology has upsurged in the medical field as a solution for treatment of rare genetic disorders. In beta-thalassemia, zinc finger protein (ZF)-based epigenome editors were fused to epigenetic modifiers to achieve activation of specific endogenous genes and modulate the gene expression. The limitation of using the zinc finger-based editors is their low specificity and binding to off-target sites.^29^ Hence, other epigenome editing platforms with higher DNA recognition capacities play a crucial role in the stable regulation of gene expression, namely Transcription activator-like effectors (TALE) and CRISPR-Cas9. However, they still have limitations. Comparatively, CRISPR-Cas9-based epigenome editors present several advantages over TALE and ZFs. With the use of only one Cas9 enzyme, the CRISPR-Cas9 system facilitates simultaneous epigenome editing of multiple regions.^30^ Several studies were conducted to investigate the beta-globin gene regulation mechanism using artificial transcription factors and epigenome editors to reactivate human gamma- or beta-globin gene expression.^31^, ^32^, ^33^, ^34^ To step into therapeutics, several paces are needed to be taken care of for the usage of epigenome editing, and it is necessary to develop protocols for the delivery system.

### Non-coding RNA in thalassemia

The non-coding RNA (ncRNA) is a functional RNA molecule and constitutes a heterogeneous group of transcripts not translated into proteins. The two major types of ncRNA are small RNA (sRNA) and long non-coding RNA (lncRNA). sRNA are important regulatory molecules in the control of gene expression at both transcription and post-transcriptional level by gene silencing or RNA silencing.^35^ The types of sRNA include microRNA (miRNA), small interfering RNA (siRNA), small nuclear RNA (snRNA), small nucleolar RNA (snoRNA) and piwi-interacting RNA (piRNA) which are majorly involved in regulating various biological processes. lncRNA, including intergenic, intronic, sense and antisense lncRNAs, are reported to be the most prevalent and functionally diverse members of ncRNA.^36^ The role of lncRNA can be perceived in gene expression, genomic imprinting, nuclear organization, gene dosage compensation, chromatin structure modulation, RNA translation, splicing and epigenetic regulation.^37^

The role of epigenetic regulators and modifiers, including lncRNA in hemoglobin synthesis and the role of dysregulated lncRNA have been studied extensively, but their role in changing the expression of the human globin gene has not been studied in depth.^38^ In normal cells, lncRNA prevents the binding of miRNA to maintain the Hb F levels, whereas in disease conditions like beta-thalassemia, due to dysregulated lncRNAs the level of Hb F is elevated.^39^ The possible mechanism for high levels of Hb F is the activation of Hemoglobin Subunit Epsilon 1 (HbE1) and haemopoietic cell lineage-inducible molecule by lncRNA. Several studies have reported various mechanisms of regulation of lncRNA in the expression of the gamma-globin gene.^40^, ^41^, ^42^, ^43^ The lncRNAs, like Metastasis-Associated Lung Adenocarcinoma Transcript 1 (MALAT1), Myocardial Infarction Associated Transcript (MIAT), Antisense Non-coding RNA in the Inhibitors of cyclin-dependent kinase 4 Locus (H19 and ANRIL), are differentially expressed in beta-thalassemia, thereby acting in a putative role in beta-thalassemia pathophysiology.^43^^,^^44^

Apart from lncRNA, miRNA plays a major role in hemoglobinopathies, such as regulating gene expression, erythroid cell mechanism, iron hemostasis, and oxidative cell damage. *miR15a/16–1, miR-486–3p, miR-26b, miR-199b-5p, miR-210, miR-34a, miR-138, miR-326, let-7, and miR-17/92* cluster elevate gamma-globin expression, whereas *miR-451* induces alpha-, beta- and gamma-globin expression.^45^ Down regulation of the circulating miRNAs *miR-let-7d, miR-200b* and up-regulation of *miR-122* in TDT can serve as biomarkers for cellular damage under excessive iron conditions in tissue.^46^ In a recent study, Penglong et al.^47^ showed a biphasic expression of *miR-214* in beta- and alpha-thalassemia and the molecular mechanism of miRNA and transcription factors in the regulation of oxidative status in erythroid cells in thalassemia.

### Role of artificial intelligence in thalassemia

The simulation of human intelligence using machines to generate, classify and perform cognitive functions through technology is called artificial intelligence (AI). The use of AI has increased in the field of healthcare for accurate and swift diagnosis of disease.^48^ Several machine learning algorithms play a key role in diagnosing and differentiating thalassemia from iron deficiency anemia.^49^ AI-based tools are required to predict the prevalence of genetic mutations in thalassemia much earlier, before expending more on diagnosis and treatment.^50^ It is essential to have collaboration between engineers and healthcare practitioners to decide on the development of algorithms and models to solve problems using specific knowledge and approaches to improve the quality of life for patients.

### Molecular diagnostics demand for thalassemia

Though different molecular approaches exist for the detection, screening and diagnosis of thalassemia their utilization in routine clinical practice is limited by socioeconomic conditions and by the awareness of the patients and their relatives. At the same time, in-depth knowledge about the currently available techniques, along with the advantages and limitations of the same, is important to choose the correct testing methodology applicable to concerned tertiary healthcare facilities and the patients attending them. NGS is widely available in the market, and because of the numerous publications, all departments are aware of its existence and usage. Broader panels including multiple genes or shorter panels with the targeted genes, which have clinical implications prevalent in our population, can be studied using the same technique. Quantitative polymerase chain reaction (qPCR) is another more sensitive methodology when compared to NGS, as the results obtained from NGS during a research protocol are always validated using qPCR. qPCR is a cost-effective methodology that can be used in diagnostics and hence can be used with increased sensitivity for thalassemia mutation testing, both in patients and also in instances of prenatal screening. Testing of the *HBB* gene using the qPCR technique is capable of detecting the most common mutations known to occur in the Indian population. Using the NGS technique, additional mutations (both prevalent and non-prevalent) in the *HBB* gene can be covered with less sensitivity. This pitfall of reduced sensitivity is associated with the errors and false-positive data that can occur as part of data analysis. Expertise is needed for pre-analytical, analytical and post-analytical procedures, since error-free performance of the technique is not widely available everywhere, especially in resource-poor settings.

## Conclusion

Although various molecular approaches exist to detect and treat thalassemia, the burden of the disease is increasing worldwide. It is necessary to create widespread awareness and adopt diagnostic and prenatal screening programs to provide appropriate supportive care and treatment for affected patients and, at same time, to prevent the birth of affected babies. The testing methodology adopted for this should be cost-effective, sensitive, specific and easy to perform in resource-poor settings. Based on the extensive literature review available, it is suggested that qPCR may be considered a viable option for thalassemia mutation testing of the Indian population. NGS may be reserved for a clinically suspected thalassemia patient who does not harbor a detectable mutation by qPCR. Moreover, qPCR is sufficient to detect mutations in prenatal screening. In summary, this review aimed to discuss multiple molecular-level approaches to detect mutations in thalassemia and therapeutic knowledge about the application of gene editing technologies in the treatment of thalassemia. It also discusses the epigenetic mechanisms and the role of non-coding RNA which may serve as a biomarker for disease diagnosis. The need for molecular testing diagnostics is warranted, and it should, at the same time, be made affordable.

## Conflicts of interest

The authors declares no conflict of interest.
